# IgG4-Related Kidney Disease: A Diagnostic Conundrum Successfully Treated With Steroids and Rituximab

**DOI:** 10.7759/cureus.52000

**Published:** 2024-01-10

**Authors:** Anand Chellappan, Amol Bhawane, Alok Sharma, Ratnesh Rokade

**Affiliations:** 1 Nephrology, All India Institute of Medical Sciences, Nagpur, Nagpur, IND; 2 Renal Pathology, Dr. Lal PathLabs/National Reference Lab, New Delhi, IND

**Keywords:** igg4-related kidney disease, retroperitoneal fibrosis, rituximab, steroids, immunoglobulin g4-related disease

## Abstract

IgG4-related disease (IgG4-RD) is an immune-mediated fibroinflammatory condition that has been recognized as a unified systemic disease that links many individual organ conditions that were previously considered to be unrelated. The pathological hallmark of the disease is an abundant IgG4-positive plasma cell infiltration in the affected tissues and fibrosis. It is a great mimicker of neoplastic, inflammatory, and infectious conditions. We report a 72-year-old man who presented to our hospital with dyspnea and oliguria. Detailed evaluation revealed that he was treated at multiple places for right-sided loin pain over the past 10 months and was found to have right-sided hydronephrosis, renal dysfunction, and multiple enlarged lymph nodes. A search for underlying malignancy previously was unyielding and he had rapid worsening of renal function prior to the current presentation. He was uremic and was initiated on hemodialysis. Kidney biopsy revealed features of IgG4-related tubulointerstitial nephritis. Despite tubular atrophy and interstitial fibrosis involving more than 50% of the sampled cortex, he showed a good response to steroids and rituximab (RTX) and became dialysis-independent. This report underscores the masquerading presentation of IgG4-RD which can hinder timely diagnosis and demonstrates the usefulness of a regimen of steroids and RTX in its treatment.

## Introduction

IgG4-related disease (IgG4-RD) is an immune-mediated fibro-inflammatory disorder that has been recognized as a unified systemic disease over the past two decades. It was first described in a patient with sclerosing pancreatitis with an elevated serum IgG4 level in the year 2001 [1]. It brings together several conditions that were previously believed to be unrelated - Type 1 autoimmune pancreatitis, sclerosing cholangitis, retroperitoneal fibrosis (RPF), hypertrophic pachymeningitis, Mikulicz’s disease, and Riedel’s thyroiditis [2]. The pathological hallmark of the disease is a dense lymphoplasmacytic infiltrate in the affected tissues with IgG4-positive plasma cells and fibrosis. It often obscures diagnosis since clinical, laboratory, and imaging studies fail to distinguish it from inflammatory, malignant, and infectious diseases. We report a 72-year-old man with a nearly 10-month undiagnosed condition, who presented with rapidly progressive renal failure due to IgG4-related kidney disease (IgG4-RKD). Successful treatment with steroids and rituximab (RTX) rendered him dialysis-independent.

## Case presentation

A 72-year-old man presented with NYHA (New York Heart Association) class IV dyspnea and decreased urine output for two weeks. He was a type II diabetic and hypertensive for 10 years with no retinopathy. He was asymptomatic until May 2022 when he first developed right-sided loin pain and was found to have right hydroureteronephrosis and renal dysfunction (Table 1) elsewhere. 

**Table 1:** Investigations at the initial presentation SGPT: Serum glutamic pyruvic transaminase; GGT: Gamma glutamyl-transferase; CRP: C-reactive protein

Investigation	Report	Reference Range
Hemoglobin (g/dL)	10.8	13-17
Total Leucocyte Count(/µL)	6360	4000-11,000
Neutrophils (%)	75	40-75
Lymphocytes (%)	15	20-40
Monocytes (%)	8	2-10
Eosinophils (%)	2	1-6
Platelet count(/cu.mm)	477000	1,50,000-4,50,000
Serum creatinine (mg/dL)	1.98	0.8-1.3
Serum CRP (mg/dL)	76.59	1-5
Serum uric acid	5	3.5-7.2
HbA1c (%)	6	Less than 5.7
Urine routine and microscopy		
Albumin	Nil	Nil
Sugar	Nil	Nil
RBCs (/hpf)	Nil	0-2
Pus cells (/hpf)	0-2	0-5
Serum protein electrophoresis	No monoclonal band seen	No monoclonal band
Serum total protein (g/dL)	7.31	6.4-8.3
Serum albumin (g/dL)	3.75	3.57-5.42
Serum IgG total (mg/dL)	2018	700-1600
Serum IgA total (mg/dL)	139	70-400
Serum IgM total (mg/dL)	20.1	40-230
Serum beta 2 microglobulin (ng/mL)	12090	670-2143
SGPT (U/L)	25	7-56
Serum GGT (U/L)	36	9-48

The computerized tomography (CT) scan revealed a soft tissue density encasing the aorta extending from the aortic bifurcation to the origin of the renal arteries with right-sided hydronephrosis. Multiple sub-centimetric and few enlarged lymph nodes were noted in the pre- and para-aortic regions. He had undergone retrograde pyelography and double J (DJ) stenting in November 2022. In view of multiple enlarged lymph nodes, he had undergone evaluation for underlying malignancy (CA 19-9, CEA, PSA, and serum protein electrophoresis were negative) and the FDG-PET scan revealed hypermetabolic hypodense lesions in bilateral testis, cervical, mediastinal, retroperitoneal, pelvic, and left inguinal lymph nodes. Right orchidectomy done elsewhere revealed mild interstitial lympho-plasmacytic inflammatory infiltrate with no evidence of malignancy. He had progressive renal dysfunction over the four weeks prior to the current presentation. The serum creatinine had worsened from 3.9mg/dL to 9.1mg/dL (Table 2) despite the treatment offered elsewhere and he presented to us with oliguria and dyspnea. He was uremic and dyspneic for which he was started on hemodialysis. 

**Table 2:** Investigations at the current presentation EBV: Epstein–Barr virus

Investigation	Report	Reference Range
Hemoglobin (g/dL)	7.8	13-17
Total leucocyte count (x10^3^/ µL)	7.26	4-10
Platelet count (x10^3^/ µL)	231	150-450
Serum creatinine (mg/dL)	9.1	0.6-1.3
Serum uric acid (mg/dL)	6	2.6-6
Serum total protein (g/dL)	7.9	6.4-8.3
Serum albumin (g/dL)	3.6	3.2-4.5
Serum calcium (mg/dL)	8.6	8.4-10.2
Serum phosphorous (mg/dL)	7.9	2.5-4.5
HbA1c (%)	6	Less than 5.7
Urine routine and microscopy		
Albumin	Nil	Nil
Sugar	Nil	Nil
RBCs (/hpf)	6-8	0-2
Pus cells (/hpf)	15-20	0-5
Urine culture	No growth	No growth
EBV quantitative blood PCR	Target Not Detected	Lower limit of detection: 40 copies/mL

**Figure 1:**
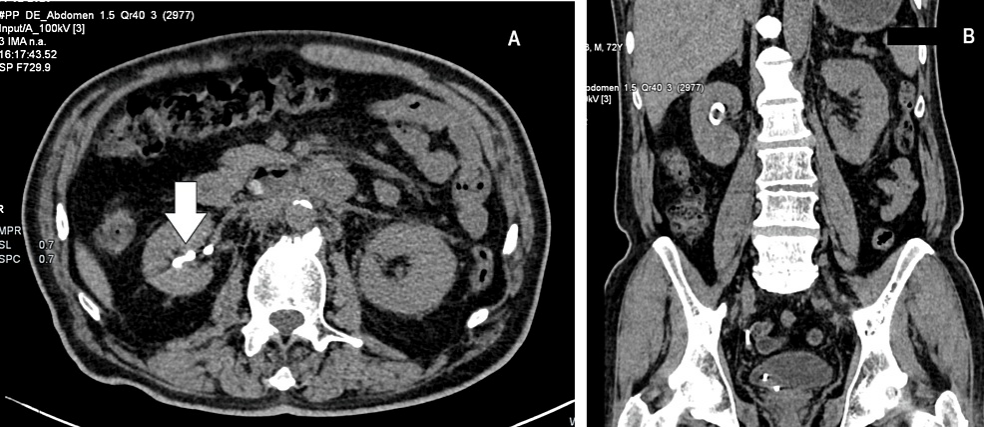
Computerized tomography scan images (A: axial view; B: coronal view) showing right-sided double J stent (white arrow) in situ and bilateral perinephric fat stranding

The computerized tomography (CT) scan (Figure 1) revealed a right kidney of size 6.6x4.1x8.5cm with DJ stent in situ. There was no calculus or hydronephrosis. The left kidney was 5.7x8x10.6cm in size with no calculus or hydronephrosis. There was mild perinephric fat stranding noted on both sides. A review of the orchidectomy histopathology specimen with IgG and IgG4 immunohistochemistry did not reveal an excess of IgG4-expressing plasma cells.

**Figure 2:**
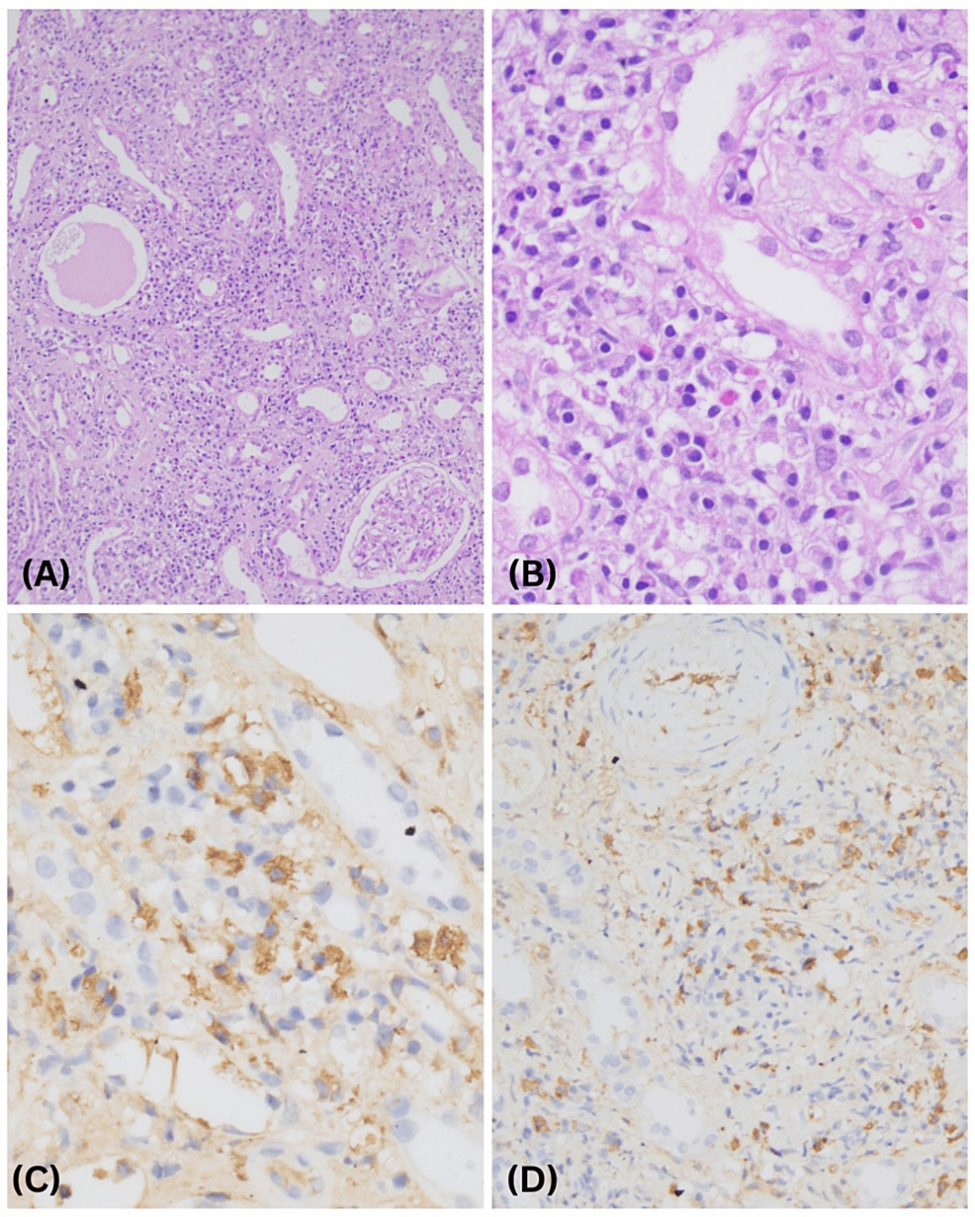
Kidney histopathology (A) Low magnification: unremarkable appearing glomerulus and dense interstitial inflammation in background. (B) Light microscopy: focal dense chronic interstitial inflammation composed of many plasma cells, lymphocytes, histiocytes, and scattered eosinophils. (C & D) Immunohistochemistry: interstitial inflammatory infiltrate composed of >10 IgG4 positive plasma cells/hpf

Kidney biopsy was done in view of the rapid worsening of renal function (Figure 2). It revealed 10 glomeruli, none sclerosed, with focal capillary congestion and focal periglomerular fibrosis, with no significant proliferative or exudative activity. IFTA involving more than 50% of the sampled cortex with prominent focal interstitial inflammation with plasma cells, lymphocytes, histiocytes, and scattered eosinophils was noted. Arteries showed medial thickening and fibrointimal sclerosis, while arterioles revealed focal marked hyalinosis. The glomerular immunofluorescence staining was negative, but focal granular staining for IgG, Kappa, and Lamba light chains was noted along the tubular basement membrane. Immunophenotyping of interstitial inflammatory infiltrate revealed >10 IgG4 positive plasma cells/hpf in the most concentrated field. The serum IgG4 level was more than 3.93g/L.

**Figure 3:**
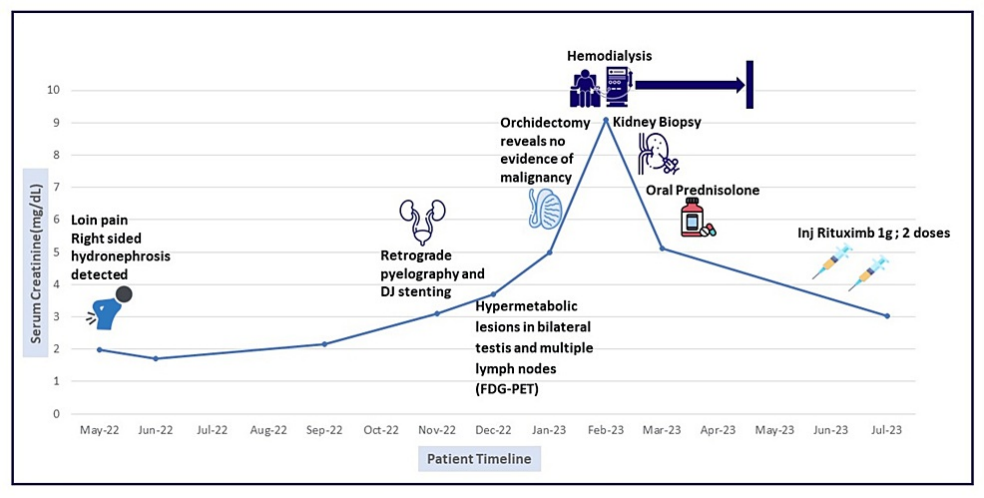
Patient timeline depicting the chronology of events DJ: Double J; FDG: fluorodeoxyglucose, PET: positron emission tomography

He was diagnosed with IgG4-RKD and was started on 1mg/kg of oral prednisolone for three weeks and tapered over three months. Two doses of RTX (1g each; 15 days apart) were given. His renal functions improved progressively. He required 10 sessions of hemodialysis following which he became dialysis independent. DJ stent was removed. Prednisolone was tapered and stopped. After five months of follow-up, he has a serum creatinine of 3.02mg/dL and a urine protein creatinine ratio of 279mg/g and is asymptomatic. The patient timeline is depicted in Figure 3.

A regular monthly follow-up is planned for the patient in view of the chances of recurrence. We plan to follow up on this patient with the following investigations: A. Complete blood count with differential, kidney function test, urine routine and microscopy, urine spot protein creatinine ratio, and CRP levels every month, B. Serum IgG4 levels every 3-6 months, and C. Annual ultrasound imaging of the kidneys and ureters. The patient shall be examined clinically during every visit for evidence of relapse and other organ involvement. At present, the patient is off steroids. To prevent relapses, a maintenance regimen consisting of six monthly infusions of 1g of RTX is planned. 

## Discussion

IgG4-RD is a fibro-inflammatory disease with an elevated serum IgG4 level and marked tissue infiltration of IgG4-positive plasma cells which brings together several conditions that were previously believed to be unrelated. IgG4-RD is a great mimicker and often evades diagnosis as in our case who was suspected to have an underlying malignancy and remained undiagnosed for more than six months. He had periaortitis, RPF with hydronephrosis, and an elevated serum IgG4 level. Diagnostic criteria for IgG4-RKD have been developed and validated [3]. IgG4-RKD encompasses renal parenchymal and pelvic lesions. The frequency of kidney involvement in IgG4-RD ranges from 7% to 24.6%. Tubulointerstitial nephritis is the most common manifestation. The typical pathological finding of IgG4-positive plasma cells >10/high power field was seen in our patient. Eosinophilic infiltration, ‘storiform fibrosis’, and ‘obliterative phlebitis’, and among the glomerular lesions, membranous nephropathy are important features of IgG4-RKD which were not found in our case. RPF/periaortitis has been reported in 9.6%-27% of the cases of IgG4-RD and hydronephrosis in 33-67% of cases, of which nearly 75% is unilateral [4]. A close mimic of IgG4-RD is multicentric Castleman’s disease. The normal serum IgA level and prompt response to steroids differentiated it from IgG4-RD in our case.

The international consensus guidance statement on the management and treatment of IgG4-RD recommends glucocorticoids as the first-line treatment for treatment induction in active untreated IgG4-RD in the absence of any contraindications [5]. The typical starting doses of 0.6-1mg/kg body weight are given for 3-4 weeks and tapered over 3-6 months. A prompt response to steroids differentiates it from other diagnoses. Our patient had a prompt response to steroids making him dialysis independent. The extent of recovery of renal functions depends on the timing of initiation of steroids. Saeki et al. found that patients with an eGFR of less than 60ml/min at the time of starting steroids had a significant recovery of renal function [6]. However, the recovery was only partial. A delay in the institution of steroids explains the incomplete recovery of renal function in our patient. Apart from steroids, B-cell depletion with RTX appears to be a promising therapy for induction and maintenance in IgG4 RD. Quattrocchio et al. found that a single course of intensive B-cell depletion therapy was safe and effective [7]. Carruthers et al. found that 97% of the 30 patients with IgG4-RD responded well to two doses of RTX (1000mg each) [8]. It included seven patients with renal lesions.

IgG4 RD has a relapsing-remitting course necessitating a maintenance regimen. Patients with multi-organ involvement, elevated serum IgG4 and IgE levels, and peripheral eosinophilia show the highest risk of relapse. Maintenance therapy may consist of low-dose glucocorticoid therapy or steroid-sparing agents [9,10]. There is little evidence to guide the maintenance therapy in IgG4-related kidney disease. Glucocorticoid monotherapy may fail to adequately control the disease and also predispose the patients to adverse events on long-term use. RTX is useful as a maintenance therapy; however, the dose, frequency, and duration are not clearly defined. Maintenance therapy with RTX may be given at predetermined intervals or when there is evidence of a disease flare. A nationwide multicenter study in France found that systematic administration of RTX is associated with a longer relapse-free survival [11]. Another study found that a single infusion of RTX 1g given every six months is equally effective in maintaining disease remission as two doses of RTX 1g administered 15 days apart [12]. Our patient is currently off steroids. A maintenance regimen with 1g of RTX administered every six months is planned. 

This case highlights several important aspects of IgG4-RKD. It portrays the difficulty and delay in achieving the diagnosis of IgG4-RD. It underscores the importance of timely diagnosis and institution of therapy and provides evidence of the usefulness of a regimen comprising steroids and RTX in the management of IgG4-RKD. 

## Conclusions

IgG4-RKD masquerades and eludes timely diagnosis, underscoring the need for prompt recognition. Initiating steroids and/or RTX promptly, before significant fibrosis develops, is associated with good outcomes.
